# How Many Alzheimer–Perusini’s Atypical Forms Do We Still Have to Discover?

**DOI:** 10.3390/biomedicines11072035

**Published:** 2023-07-19

**Authors:** Luigi Donato, Domenico Mordà, Concetta Scimone, Simona Alibrandi, Rosalia D’Angelo, Antonina Sidoti

**Affiliations:** 1 Department of Biomedical and Dental Sciences and Morphofunctional Imaging, Division of Medical Biotechnologies and Preventive Medicine, University of Messina, Via Consolare Valeria 1, 98125 Messina, Italy; ldonato@unime.it (L.D.); domenico.morda@studenti.unime.it (D.M.); cscimone@unime.it (C.S.); rdangelo@unime.it (R.D.); asidoti@unime.it (A.S.); 2 Department of Biomolecular Strategies, Genetics, Cutting-Edge Therapies, Euro-Mediterranean Institute of Science and Technology, Via Michele Miraglia, 98139 Palermo, Italy; 3 Department of Chemical, Biological, Pharmaceutical and Environmental Sciences, University of Messina, Viale Ferdinando Stagno D’Alcontres 31, 98166 Messina, Italy

**Keywords:** atypical AD forms, microglia, genetics, amyloid plaques, neurofibrillary tangles

## Abstract

Alzheimer–Perusini’s (AD) disease represents the most spread dementia around the world and constitutes a serious problem for public health. It was first described by the two physicians from whom it took its name. Nowadays, we have extensively expanded our knowledge about this disease. Starting from a merely clinical and histopathologic description, we have now reached better molecular comprehension. For instance, we passed from an old conceptualization of the disease based on plaques and tangles to a more modern vision of mixed proteinopathy in a one-to-one relationship with an alteration of specific glial and neuronal phenotypes. However, no disease-modifying therapies are yet available. It is likely that the only way to find a few “magic bullets” is to deepen this aspect more and more until we are able to draw up specific molecular profiles for single AD cases. This review reports the most recent classifications of AD atypical variants in order to summarize all the clinical evidence using several discrimina (for example, post mortem neurofibrillary tangle density, cerebral atrophy, or FDG-PET studies). The better defined four atypical forms are posterior cortical atrophy (PCA), logopenic variant of primary progressive aphasia (LvPPA), behavioral/dysexecutive variant and AD with corticobasal degeneration (CBS). Moreover, we discuss the usefulness of such classifications before outlining the molecular–genetic aspects focusing on microglial activity or, more generally, immune system control of neuroinflammation and neurodegeneration.

## 1. Introduction

About 120 years have now passed since the neuropsychiatrist Alois Alzheimer visited Auguste Deter, a 50-year-old woman, who presented loss of memory and very strange behavior. Afterwards, the Italian neurologist Gaetano Perusini described three patients (47, 63 and 67 years of age) [1]. These were the first cases of Alzheimer–Perusini’s disease [2] that today represents the most widespread type of dementia around the world. It is estimated that over one million people suffer from dementia in Italy, including 600,000 with Alzheimer’s, and about 3 million people are involved directly or indirectly in their assistance, with additional consequences on an economic and organizational level. The phenomenon is increasing due to the aging of the population. According to the demographic projections reported by the Italian Ministry of Health website and based on current trends, in 2051, there will be 280 elderly people for every 100 young people, with an increase in all age-related chronic diseases, including dementia [3]. Age remains the most important risk factor for this pathology [4] in spite of the above-mentioned cases who were younger. What progress has been made in recent years in understanding the disease and patient care? Alzheimer, Perusini and colleagues described well the histopathological hallmarks compatibly with the means of the time: at a macroscopic level, marked cerebral atrophy with enlarged sulci and constricted gyri; at a histologic level, neurofibrillary tangles, neuritic plaques and a strange substance present in extracellular space and near vessels (afterward recognized as β-amyloid). Now, it seems that very few (or probably zero) cases present with just these alterations; usually amyloidosis is accompanied by multiple proteinopathies [5] (some people read this pathological picture as a series overlapping with other neurodegenerative pathologies), such as hyperphosphorylated tau protein, TDP-43, α-synuclein or the prion protein. Moreover, there is a significant synaptic loss, in particular cholinergic (this is the reason why one of the few pharmacological classes approved for AD is anticholinesterases [6]). However, in a small group of cases, levodopa administration was approved because of dopaminergic synapse [7] loss. The synaptic destruction is due to the synergistic effect of tau and Aβ [8]. Above all, AD etiopathogenesis seems to originate from immune system alterations [9] and microbiota dysbiosis [9], and going deeper, the first trigger can be the altered transition metal ion allostasis with consequent mitochondrial disfunction. These abnormalities can arise during nervous system (NS) development. Corroborating this assertion, an altered PAX6 pathway in AD individuals [10] (similar to what happens in certain retinal dystrophies, where PAX2 is closely related to PAX6 [11]) was described and TREM2 (a microglial triggering receptor, also involved in amyloid phagocytosis) mutations are supposed to be involved in autism [12]. In the epigenetic field, a particular modification, hydroxymethylation, was found to be closely related to brain function and neurodegenerative disease. Two of three hydroxymethylating enzyme (TET) isoforms, TET2 and TET3 [13], seem to be actors in various neurodegeneration pathways. Also, RNAi has been intensively studied in recent years; for example, in a series of Aβ synthetic pathway regulatory RNAs, such as BACE1-AS and mir485-5p [14], and the smallest of the known lncRNAs, BC200. This is able to regulate district-specific translation in neurons, binding and trafficking the mRNA–ribosome complex [15]. Surely, we can consider AD as a multifactorial disease where together with the above-mentioned alterations genetic background and environmental influence can strongly contribute to risk. All these co-causes can only result in a wide spectrum of different phenotypes that today is called the “Alzheimer continuum” [16]. A lot of work has demonstrated that the symptoms follow a tau distribution in the brain: areas affected by NFT become dysfunctional. This leads to a very large symptomatologic spectrum. It is common to distinguish a typical amnestic form from atypical ones. The former is defined as LOAD (late-onset AD); the latter are early-onset forms, and of those just one is characterized by the Mendelian inheritance (bound to three causative genes: APP, PSEN1 and PSEN2), while the others are related to a gene panel and internal and external environmental influence. On the basis of the symptoms (and so NFT-stricken brain areas), we can discern four main atypical forms: posterior cortical atrophy (PCA), logopenic variant of primary progressive aphasia (LvPPA), frontal AD and AD with corticobasal syndrome (AD with CBS) [17]. More details are available in the next section. What definition could we provide for this complex pathological scenario? The DSM-V (American Psychiatric Association Diagnostic and Statistical Manual) answers: “Major neurocognitive disorder due to Alzheimer’s disease”, where “AD is a gradually progressive neurodegenerative disorder characterized by the progressive deposition of amyloid plaques and neurofibrillary tangles in the cortical brain tissue” [18]. It is not an informative definition, but it is probably true that a huge number of recent findings are not useful on the clinical side, up to now. However, they might be soon. Some recent articles coined the word “immunoproteostasis” [19], recognizing a key role of the glial cells in triggering proteinopathies and pathology. This might occur because altered glia can constitute the fundamental difference between neurodegenerative pathology and slow neurodegeneration due to normal or paraphysiological aging. When the correct name is assigned to something (definition is provided), it means that it is understood, and if it is understood, it can be hypothesized about, specifically how to alter it and its natural history. Hence, when we understand all or almost all the secrets of this complex molecular picture, we will manage to find a disease-modifying therapy (DMT), a few “magic bullets” acting synergistically and accompanied by lifestyle changes [20]. Aducanumab was the first drug approved for Alzheimer–Perusini’s disease in 2019 [21]. It is an mAb directed against β-amyloid plaques that is able to pass the hematencephalic barrier (HEB) [22]. In preclinical studies, it seemed to efficiently reduce the amount of Aβ; however, its administration has been limited because of some severe adverse reactions such as cerebral inflammation and edema [23]. C. Humpel in the past showed that intranasal neprilysin (an enzyme capable of degrading Aβ) treatment reduced β-amyloid amount and symptoms for only a short time window, probably slowing the progression, but not solving the molecular pathology [24]. Thus, we can envision a similar situation for aducanumab that can be compounded by classic adverse events of mAb. Recently, the FDA approved therapy with lecanemab, which provides passive immunization against β-amyloid plaques, especially during early stages of the pathology [25]. Additionally, if microglia are dysfunctional, a triggering mAb might make minimal changes. A better understanding of the disease might also allow us to overcome the incredible difficulties of reaching the correct diagnosis, bringing us closer to an early pre-clinical one. Even today, in the era of neuroimaging, we must wait for post-mortem evaluation of the affected brain to differentiate between different neurodegenerative pathologies due to significant overlaps. Certainly, we could avoid misdiagnosis by combining FDG-PET, Amyloid PET, Tau-PET, CSF biomarkers, and other neuroimaging techniques specific for other proteinopathies. Furthermore, AI and dedicated software could assist scientists [26]. However, this applies mainly to research studies, not clinical routine. In the next section, we update the recent findings regarding atypical forms, and in the conclusion, we attempt to provide a reliable, though incomplete, definition. Why might uncommon and atypical forms be significant in our investigation of the comprehensive submolecular, molecular, and supramolecular explanations for disease pathogenesis? Primarily, from a genomic perspective, these unusual cases encompass a cluster of implicated genes, which differs from the one or two usually seen in the Mendelian inheritance or the thousands present in Late-Onset Alzheimer’s Disease (LOAD), where normal aging is the dominant factor. In these specific disorders, there is established evidence of dysfunctional glial cells, prompting researchers to hypothesize about changes in the genetic makeup of immune cells during both developmental and adult stages [27]. It is crucial to understand that the fundamental unit of the nervous system (NS) comprises three elements: a neuron, a vessel, and a glial cell. Therefore, if the glial cell is dysfunctional, this aberration would inevitably affect the other two elements to varying degrees, depending on different development phases and brain regions. Importantly, this shift in focus from neurons to microglia represents a significant perspective change and a major challenge, tantamount to a Kantian revolution in neuropathology.

## 2. A Sea of Atypical Forms Classification: Let Us Dive in!

### 2.1. Classification Based on Regional NFT Density

**Figure 1:**
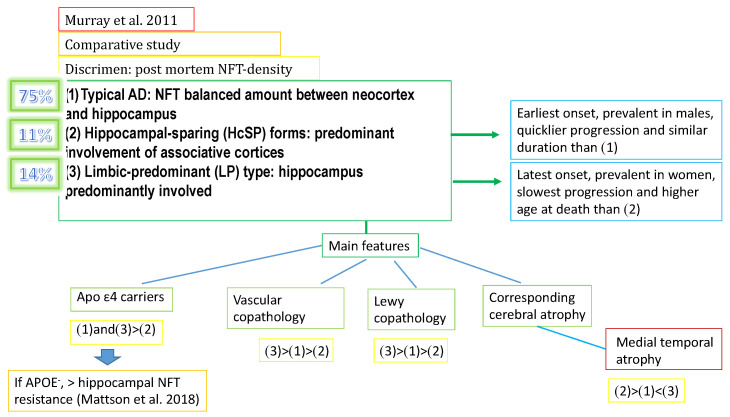
Murray et al. [29] classification and its features.

Along with ADNC (Alzheimer’s disease neuropathological changes), while Aβ is usually diffuse, the NFTs are concentrated in specific brain areas. These NFT-affected areas show impaired function; hence, tau pathology is directly related to the range of symptoms [27]. In particular, it seems to follow specific trajectories in synaptic networks during its progressive distribution [28]. Based on these findings, NFT distribution patterns could be adopted as a criterion to draft a preliminary classification. For example, Murray et al. [29] in 2011 carried out a comparative study that relied on postmortem NFT density in the AD-brain. This led to the distinction of three main subdivisions: (1) typical AD (tAD), (2) hippocampal-sparing (HcSP) forms, and (3) the limbic predominant (LP) type. TAD showed a balanced amount of NFTs between the neocortex and hippocampus and represented 75% of patients. HcSP forms predominantly involved associative cortices; they showed the earliest onset, a faster progression than tAD, and were present in 11% of patients. Finally, the LP type predominantly affected the hippocampus and represented 14% of patients, primarily women. Its mean age at symptom onset was later, progression slower, and age at death higher than those of the other two groups. As far as the ApoE genotype is concerned, ε4 carriers are likely to suffer from LP or tAD: ApoE-negative individuals seem to have an ADNC-resistant hippocampus [30]. With regard to vascular co-pathology, it showed the following trend: LP-AD > tAD > HcSP-AD, while in Lewy co-pathology, it was lowest in LP-AD [31]. Several research groups [32] have investigated the corresponding atrophic profiles via MRI: LP-AD > tAD > HcSP-AD in the medial temporal lobe, and HcSp-AD > tAD > LP-AD in cortices (Figure 1).

### 2.2. Classifications Based on Neuroimaging Studies

**Figure 2:**
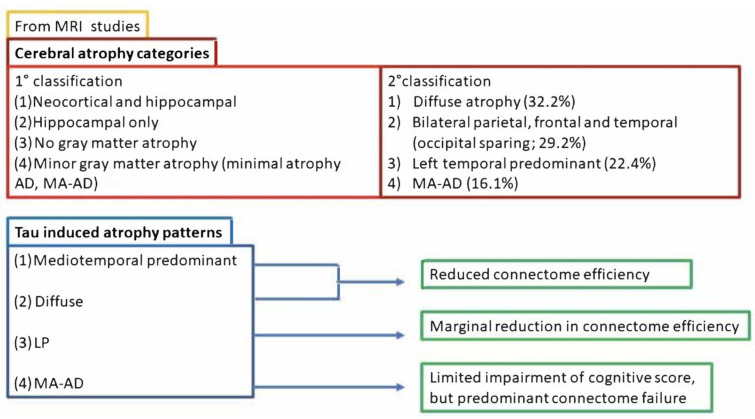
Two-atrophy pattern [32,40] and tau-induced atrophy pattern [40] classifications.

However, numerous other studies have analyzed cerebral atrophy patterns through MRI analysis, allowing the establishment of two main classifications as reviewed earlier by K.A. Jellinger in 2021 [26]. The first recognizes these four subtypes: (1) both neocortical and hippocampal; (2) hippocampal only; (3) minor gray matter atrophy; and (4) no gray matter atrophy [33,34,35,36]. Subtypes (3) and (4) exhibit comparable clinical severity and are characteristic of minimal atrophy AD (MA-AD). MA-AD presents a specific CSF biomarker scenario, with increased ptau [37] and decreased Aβ [38]; moreover, it has been proven that it increases the risk of AD development over time [39]. The second classification is based on the study by Zhang et al. published in 2021 [39], which categorizes patients into four subdivisions: (1) diffuse atrophy (32.2%), bilateral parietal, frontal, and temporal atrophy (occipital sparing, 29.2%), left temporal dominant (22.4%), and MA-AD (16.1%) (Figure 2). 

**Figure 3:**
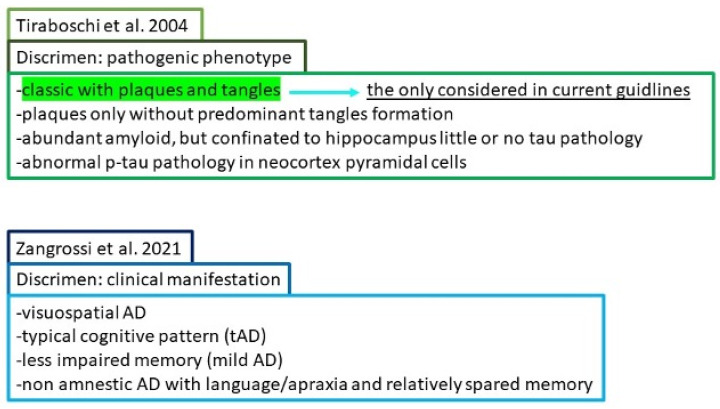
Recognized AD pathogenic phenotypes [45] and a classification reliant on clinical manifestation [46].

The same reasoning can be applied to the patterns of cerebral atrophy in prodromal AD, as summarized in two recent works. From the former [33], we can extrapolate a largely normal neuroanatomical profile with minimally abnormal cognitive and CSF biomarker patterns and the slowest progression; a classic AD form with the fastest progression; a diffuse atrophy profile, albeit milder in the medial–temporal lobe and with worse cognitive deficits; and finally a notable focal involvement of the medial–temporal lobe with a slow but steady rate of progression. Regarding the latter [41], we can recognize four other subtypes: (1) medial–temporal predominant atrophy with significant memory and language impairment; (2) parieto-occipital atrophy with poor executive/attention and visuospatial functioning; (3) mild atrophy with the best cognitive performance and language; (4) diffuse cortical atrophy with intermediate cognitive functions (visuospatial function is the first to decline). Rauchmann et al. in 2021 [42] grouped affected individuals into four NFT-related atrophy patterns associated with destruction of brain connections (Figure 2). Of these, medial–temporal predominant and diffuse patterns are accompanied by a reduction in the global efficiency of the connection networks, while LP and MA-AD are characterized, respectively, by marginal global reduction in connections and limited impairment in cognitive scores. However, in the latter case, there is significant global network failure. Interestingly, three hypometabolic subtypes were identified by sorting based on FDG-PET data [43]: (a) tAD (48.6%, classic posterior–temporal–parietal profiles); (b) “limbic predominant” (44.6%, old age and memory-predominant cognitive pattern); (c) “cortical predominant” (6.8%, relatively rare, younger age and severe executive deficit). Additionally, further progress can be made by combining MRI, tau PET, and amyloid PET. Hence, we can distinguish three AD subtypes: (a) medial temporal dominant, 53% of cases; (b) parietal dominant, 23% of cases; and (c) diffuse, 24% of cases. Aβ deposition does not change across these subtypes, suggesting a typical NFT spreading pattern starting from the entorhinal cortex and then continuing into association cortices [44]. According to this view, MA-AD should represent the earliest manifestation of the pathology, which initially degenerates into LP-AD and, finally, into tAD [26]. Due to the variability of the plaque/NFT ratio, it is possible to distinguish different pathogenic phenotypes: the classic plaque and tangle profile, which is the only one considered by the guidelines, plaques only without predominant tangle formation, abundant amyloid, little or no tau pathology (ptau limited to the hippocampus), and abnormal ptau pathology in neocortical pyramidal cells (3.8% of dementia-affected individuals over 85 years of age) [45] (Figure 3). 

Other specific neurodegenerative conditions are uncertainly included in the AD continuum because of their overlap with other pathologies or, in general, the difficulty in classifying their pathological scenarios. 

### 2.3. Other Particular AD Atypical Forms

**Figure 4:**
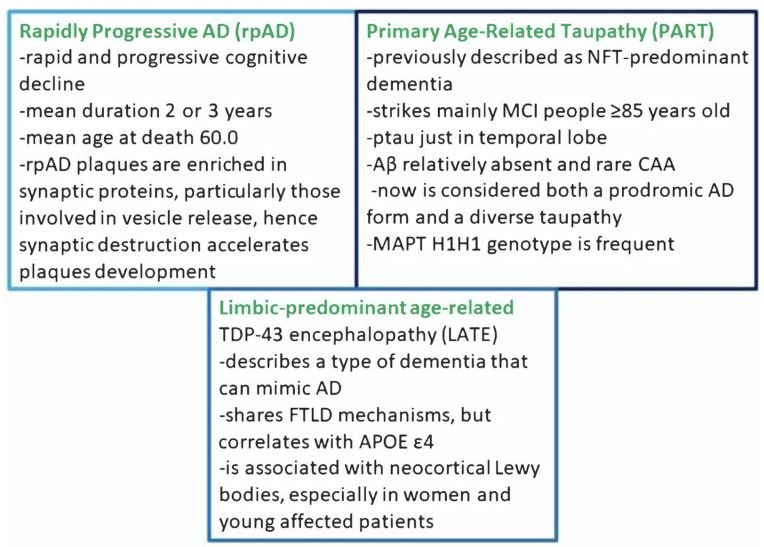
rpAD, PART and LATE main characteristics.

Now, we briefly describe rapidly progressive AD (rpAD), primary age-related tauopathy (PART), and limbic predominant age-related TDP-43 encephalopathy (LATE) (Figure 4). RpAD is characterized by a rapid and progressive cognitive decline and/or short duration of the disease (average 2–3 years, with the patient usually being young at death with an average age of 60.0 vs. 81.8 [47]); in addition, focal neurological signs are present [48]. Early executive or language dysfunction is involved, as well as movement disorders, such as myoclonus (66–75% of cases) or gait impairment (66–87%), pyramidal (53–56%) or extrapyramidal (54%) signs, visual signs (for example, hallucinations in 44–62%) or psychiatric symptoms [49,50]. K. A. Jellinger in his recent review [26] argues that the proteomic scenario of AD plaques could discern between rpAD and tAD; however, in general, metabolomics, or at least lipidomics plus proteomics, data probably are decisive for the phenotype. Regardless, Drummond et al. in 2017 [51] revealed that rpAD plaques are rich in synaptic protein, especially those implicated in synaptic vesicle release. Thus, synaptic destruction could precede plaque development in rpAD. Moreover, in this particular condition, a 1.2-fold decrease in diglycosylate PrP isoforms has been reported [52]. PrP oligomers could destabilize the neuronal actin–tubulin macromolecular complex, hence contributing to rpAD’s rapid progression [53]. Similarly, some proteomic studies [54] revealed that dysregulated and displaced ASPQ (proline- and glutamine-rich protein) caused TIA-A-positive stress granule dysfunction, thus accelerating progression. The Primary age-related tauopathy (PART) was initially described as an NFT-predominant dementia [55], which affects primarily people aged 85 years or older with mild cognitive impairment (MCI). Tau pathology seems to be confined only to the medial temporal lobe, with Braak NFT stages 0–4 [56]. Instead, Aβ is relatively absent (Thal Aβ phases 0–2); neuritic plaques are totally absent, as is cerebral amyloid angiopathy [57]. 3R and 4R (repetitions in the MAPT gene [58]) are identical to those in tAD [55]. The MAPT H1H1 genotype has high frequency in PART, LP-AD, and tAD [59]. In tAD, there is none or minimal involvement of CA2, while in PART, early-stage NFT pathology in CA2 is already higher than that in CA1. However, in PART hippocampus, tau pathology distribution is asymmetric [60,61,62], and the NFT Braak stage and the TDP-43 stage and density are positively correlated [63]. According to Kaufman et al. (2018) [64], NFT pathology starts in the transentorhinal/entorhinal region instead of the locus coeruleus. Mesial temporal tau in Aβ-negative and cognitively normal individuals likely affected by PART is correlated with worse cognitive performance and greater neocortical tau burden [65]. Limbic-predominant age-related TDP-43 encephalopathy (LATE) seems to constitute another atypical AD phenotype, but it shares FTLD-TDP mechanisms [66,67,68]. Based on LATE neuropathologic changes, the recommended grading is the following: (1) amygdala only; (2) hippocampus, (3) medial frontal cortex. LATE correlates with the APOE ε4 genotype, like AD [69,70]. AD individuals with Lewy bodies and TDP-43 typically show neuropsychiatric symptoms [71]. Neocortical Lewy bodies are associated with LATE neuropathologic changes, especially in women and young people [72]. Currently, there are no reliable biomarkers for the antemortem diagnosis of LATE [73].

### 2.4. Classifications Based on Clinical Manifestations

**Figure 5:**
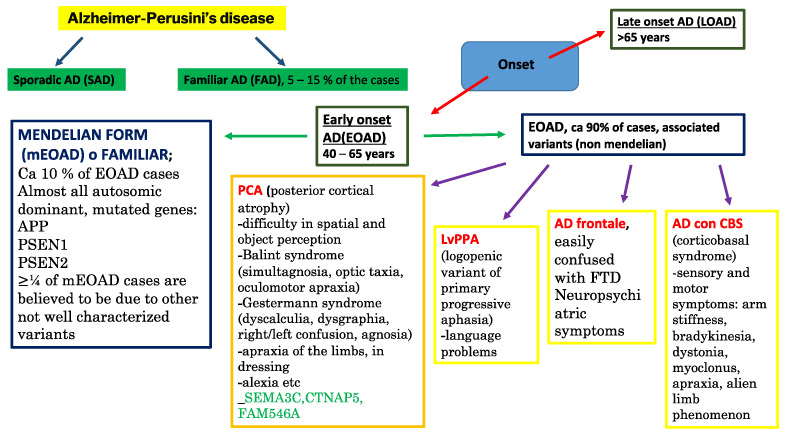
A general classification of AD phenotype, including LOAD, mEOAD and atypical forms [17].

Zangrossi et al. in 2021 [46] proposed yet another classification that relies on clinical manifestations. They differentiated four categories: Visuospatial AD; typical cognitive pattern (tAD); less impaired memory (mild AD), and non-amnestic AD with language/praxia deficits and relatively preserved memory. However, one of the most significant subdivisions we can consider is based on clinical phenotype (Figure 3). In this way, we are able to distinguish four main atypical forms: (1) posterior cortical atrophy (PCA); (2) logopenic variant of primary progressive aphasia (LvPPA); (3) frontal AD or behavioral variant/dysexecutive variant Alzheimer’s disease, and (4) corticobasal syndrome or motor variant Alzheimer’s disease [16,17] (Figure 5). 

### 2.5. PCA

#### 2.5.1. PCA Main and More Infrequent Subtypes

Crutch and colleagues recently published updated diagnostic criteria for PCA [74] and provided a classification framework that considers three PCA subtypes: PCA-pure, PCA-plus, and other pathological subtypes depending on the presentation and biomarker evidence of underlying pathology. More infrequent PCA basal pathologies include Lewy body disease, corticobasal degeneration, and Creutzfeldt–Jakob disease (CJD), the Heidenhain variant [75]. 

#### 2.5.2. Symptomatology and Clinical Features

PCA patients present with relatively good memory, language, insight, and a greater degree of posterior atrophy measured on MRI than tAD controls [76]. Affected individuals are not completely able to locate specific objects, even when they are in plain sight, and they struggle with driving (for example, they may veer out of the lane or cause accidents because they do not see objects to the side). They also show difficulty moving on uneven surfaces such as stairs and have problems positioning objects simultaneously or dressing; there is also a loss of dexterity. The progression of PCA is often insidious and primarily affects central vision without peripheral visual deficits [77]. The average diagnostic delay is about four years [78]. Not by chance, the diagnostic process can present as a grotesque story with both the patients and the clinician “looking but not seeing” [79]. Initially, patients are often referred to optometrists/ophthalmologists who may try a series of unsuccessful corrections to eyeglasses or even resort to surgery in an attempt to address the symptoms. Affected individuals may show nonspecific anxiety in the early stages of the illness. We can differentiate between dorsal, ventral, and caudal (or primary occipital) variants of PCA. The first is a biparietal form that affects the dorsal “where” visual pathway and involves visuospatial impairments, e.g., Balint syndrome (simultagnosia, optic ataxia, and oculomotor apraxia), Gerstmann syndrome (left/right disorientation, finger agnosia, dyscalculia, and dysgraphia) and limb, constructional, and dressing apraxia [74,80,81]. The second is an occipitotemporal form affecting the ventral “what” visual pathway and involves visual agnosia, apperceptive prosopagnosia, and alexia with letter-by-letter reading [82]. The last is a rare occipital form characterized by visual field defects, such as homonymous hemianopia or quadrantanopia, and a diminished visual acuity subtype [74,79,81,82]. In a recent study [83], 52 PCA patients underwent magnetic resonance imaging, fluorodeoxyglucose (FDG)-, amyloid-, and tau-positron emission tomography (tau-PET) scans, as well as a neuropsychological assessment. Only nine patients were classified as primary occipital; they were older and had more years of education than the others. This group performed worse on the Ishihara test for color perception, but better on the Western Aphasia Battery (WAB) praxis scale, and exhibited less severe neuropsychiatric symptoms compared to other affected individuals. Although FDG-PET meta-ROI was higher in caudal variant patients, no differences were noticed in amyloid- and tau-PET analysis. The authors of this work concluded that the primary features of the occipital variant are an older age at onset, more prominent color perception dysfunction, less severe ideomotor apraxia, and less hypometabolism in the temporo-parietal meta-ROI compared to other PCA phenotypes. In the early phases of PCA, insight into the pathological progression is preserved, and other cognitive domains are relatively spared [80]. In some patients, it is arduous to distinguish between CBS, LBD (Lewy body disease) and PCA because they can all manifest myoclonus, dystonia, or extrapyramidal signs, i.e., overlapping symptomatic subsets, although in a very small part of cases these are due to PCA [84]. When PCA patients attempt to copy the Rey Osterrieth complex figure, they produce a typical so-called “exploded” complex figure copy without integrating the figural components. However, the WAIS-IV Block Design, Picture Completion, and Visual Puzzles can demonstrate severe impairment in visuospatial tasks that may also manifest in the inability to write in a straight line or diminished reading capability. Visuospatial deficits might lead to impaired visuospatial memory, whereas verbal memory is usually not affected. Regarding language, it is similar to a logopenic syndrome, showing anomia, disfluency, and poor sentence repetition [51]. The recent advancements in the diagnostic field for Posterior Cortical Atrophy (PCA) can be gleaned from Crutch SJ et al.’s research [85] and are succinctly summarized in S. Manoharan and S. Munakomi’s review [86]: (1) Tau-PET imaging is deemed more specific as it accurately reflects the regions of hypometabolism and atrophy in tauopathies, providing a more precise assessment than a PET-amyloid scan; (2) The 11C-PBR28-PET scan, a microglia-specific imaging technique, exhibits enhanced binding in the occipital cortex of both hemispheres; (3) Visual Evoked Potential (VEP) testing may serve as a useful supplementary tool in diagnosing PCA. Additionally, an algorithmic approach to PCA evaluation is proposed by Maia da Silva MN, Millington RS et al. [87] which comprises the following steps:

1. Identify whether it is a neurodegenerative pathology.
2. Determine whether it is a posteriorly based cortical entity.
3. Discern whether it is pure PCA or PCA in conjunction with Alzheimer’s disease (AD), Lewy body dementia (LBD), Corticobasal Degeneration (CBD), or prion lesion.
4. Evaluate whether any relevant biomarkers such as cerebrospinal fluid (CSF) Aβ 1–42, Tau, p-Tau for AD, and 14-3-3 protein for prion disease are present.

CT or MRI in PCA individuals revealed that 96% of cases occur due to AD pathology [88]. PCA structural neuroimaging classically shows posterior-predominant atrophy with involvement of visuospatial cortices. According to a recent study [89], severe medial temporal atrophy is uncommon; mild or moderate atrophy was symmetric in 55% of individuals enrolled for the study, and in the asymmetric cluster of patients, it was usually worse on the right hemisphere (76%). Cingulate Island Sign (CIS) on [18F] fluorodeoxyglucose (FDG) PET was reported for 44% of the PCA-affected individuals (asymmetric in 50% of cases). The patients with a CIS showed more pronounced medial temporal asymmetry, but did not have lower medial temporal atrophy scores compared to those who did not show CIS. Hippocampal atrophy was found not to be bound to quantitative CIS. Hence, the previously hypothesized link between CIS and relative medial temporal sparing does not exist. In the caudal variant, the primary visual cortex shares the same pattern. Balint syndrome is associated with bilateral atrophy of the superior parietal lobule, while Gerstmann syndrome is bound to left hemisphere angular gyrus atrophy. Involvement of the right lateral parietal lobe is usually followed by such apraxia categories as dressing and constructional. In line with what we described previously, tau follows the atrophy profile, while amyloid is widespread in the brain. Finally, FDG-PET reveals posterior-predominant hypometabolism widely matching with tau distribution [18]. Older age and worse memory performance were associated with greater medial temporal atrophy. N. A. Singh, A. Arani et al. [90] assessed brain iron distribution in PCA and LvPPA patients using Quantitative Susceptibility Mapping (QSM), which works by evaluating local tissue magnetic susceptibility properties voxel by voxel. Strong evidence (posterior probability > 0.99) was found for a more pronounced susceptibility in the amygdala as well as in the middle occipital gyrus in both LvPPA and PCA and in the right inferior parietal, inferior temporal, and angular gyri in PCA only and substantia nigra and caudate nucleus in LvPPA only compared to the healthy controls. Nevertheless, moderate evidence for greater susceptibility (posterior probability > 0.90) was also reported in the precuneus, inferior occipital gyrus, entorhinal cortex, and putamen in both LvPPA and PCA, together with the superior frontal gyrus in PCA and insula, inferior temporal gyri, and basal ganglia in LvPPA compared to healthy controls. Regarding phenotypic comparison, LvPPA showed greater susceptibility in the posterior cingulate, hippocampus, and caudate; on the contrary, PCA showed greater susceptibility in the right superior frontal and middle temporal gyri. LvPPA and PCA showed moderate and strong evidence for greater susceptibility than tAD, especially in medial and lateral parietal regions, whereas tAD presents with greater susceptibility in basal ganglia and hippocampus. Therefore, a match between the distribution of iron in the brain and disease-related alterations can be observed. 

### 2.6. LvPPA

#### 2.6.1. Classifications of LvPPA and Their Symptomatology

The logopenic variant belongs to the three subgroups of Primary Progressive Aphasia and represents the one containing the largest number of estimated AD cases, 86–90%, versus 11–16% of semantic dementia and 15–20% of nonfluent/agrammatic variant PPA [88,91,92]. M. M. Mesulam, C. A. Coventry and colleagues [93] performed 118 autopsies on patients affected by PPA and diagnosed ADNC for 42% of cases, CBD or Progressive Supranuclear Palsy neuropathology in 24%, Pick’s disease (FTD spectrum) neuropathology in 10%, TDP(A) (Transactive Response DNA Binding Proteinopathy Type A) in 10%, TDP(C) (Transactive Response DNA Binding Proteinopathy Type C) in 11% and rare pathologic conditions in 3%. From these, they selected a subdivision of 68 right-handed logopenic, agrammatic/non-fluent or semantic (classified by quantitative algorithms) patients. A total of 77% of logopenics had AD neuropathologic variations, 56% of agrammatics had CBD/Progressive Supranuclear Palsy or Pick’s disease, and 89% of semantics had TDP(C) proteinopathy. Word comprehension deficits showed significant predictivity for determining underlying neuropathology, a positive correlation with TDP(C), and a negative correlation for ADNC. It is interestingly reported that one neuropathology can be responsible for multiple clinical subtypes, while one subtype of primary progressive aphasia may be caused by multiple neuropathologies, albeit with diverse probabilities. The hemispheric asymmetry of neurodegeneration and consequent language deficit in PPA (language follows dominant hemisphere) reflect complex interactions among the neurodegenerative pathology cellular affinities, the constitutive neurobiology of language cortical areas, inherited or developmental vulnerabilities of this network, and functional anatomy hypothetical idiosyncrasies in patients. Although comprehension is reduced, single-word and semantic knowledge (as measured on the Pyramids and Palm Trees Test [94]), grammatical structure, and motor speech skills are all spared in LvPPA. These findings discriminate LvPPA from the other two PPA forms; in diverse cognitive domains, overlaps reign. The paramount symptoms in LvPPA patients are word-finding troubles, circumlocution, and mispronouncing words (phonemic paraphasias). Memory dysfunction is also reported; however, elementary neurological examination turns out to be within the Gaussian bell. Delving into the specifics of speech disorders, affected individuals present word-generation problems in confrontation naming, as measured on the Boston Naming Test, verbal (animals) and letter fluency, working memory (for example, sentence repetition, digit span). Speech during image description (for example, the cookie theft picture) is relatively fluent, even if filler words and circumlocutions are common. A restricted cluster of patients can manifest language impairment only; others show a more significant cognitive deficit in verbally mediated memory tasks as measured on the Rey Auditory Verbal Learning Task or WAIS-IV Logical Memory; finally, a third group is made up of individuals with more diffuse or global cognitive impairment [95]. 

#### 2.6.2. Specific Cognitive Tests for Various PPA Forms

In a recently published commentary, J. A. Matias-Guiu and S. M. Grasso [96] summarized the latest updates on specific cognitive tests for various PPA forms. In particular, two types were described. 

(1) Patel et al. [97] dedicated themselves to the Mini Linguistic State Examination (MLSE) and showed the first validation study of it, working on a cohort of 54 PPA-affected individuals. The MLSE is a brief test which is made up of 11 subtests focused on speech and language. Five error types are registered in order to generate a deficit pattern according to motor speech, semantic knowledge, phonology, syntax, and verbal working memory. 

(2) Foxe et al. [98] recently reported the updates about the third version of Addenbrooke’s Cognitive Examination (ACE-III) utility in discriminating the PPA variants. Initially, ACE-III was developed for the assessment of frontotemporal dementia and related disorders. It examines attention and orientation, memory, verbal fluency, language, and visuospatial abilities and has now been adapted and validated in more than 30 languages. 

Nevertheless, other interesting tools for the rapid screening of PPA forms have been devised in recent years, such as the Progressive Aphasia Rating Scale (PARIS) [98] (specifically designed for PPA) and the Dépistage Cognitif de Québec [99] (a more general cognitive test for the evaluation of atypical dementias). Additionally, measures for capturing the presence/severity of language symptoms in neurodegenerative disease have been developed, for example, the Progressive Aphasia Language Scale (PALS), Progressive Aphasia Screening Scale (PASS), and the Screening for Aphasia in NeuroDegeneration Battery (SAND).

#### 2.6.3. Neuroimaging Findings

Neuroimaging findings (MRI, amyloid and tau PET, FDG-PET) reveal a widespread amyloid pattern (as already found), asymmetric atrophy (left greater than right) and a tau distribution trajectory with consequent areas of hypometabolism involving the lateral temporal and temporoparietal cortices [18]. Ramanan et al. [100] reported that the most relevant ADNC are found in the left inferior parietal lobule, left superior, middle, and inferior temporal gyri, and in the perisylvian cortical regions. A. Martersteck, I. Ayala et al. [101] demonstrated a good correlation for both Aβ and tau PET with postmortem stereological counts of amyloid plaques and neurofibrillary tangles (NFT) in a case of primary progressive aphasia (PPA) with AD neuropathologic changes, i.e., LvPPA, where atrophy asymmetrically targets the left hemisphere (the dominant one). They supported a finding in contrast with other evidence present in the literature, that is, a focal, atypical pattern of β-amyloid plaques density and florbetapir PET uptake suggests not all amyloid pathology presents as widespread in the neocortex. Some previously published works about patients affected by several types of neurodegenerations (in particular AD, because of its elevated frequency around the world) suggest that language and speech features could be exploited as a powerful screening tool. Typical tAD patients’ language has been described as “empty” with an abundance of nonspecific words, circumlocutions, and sparse content [102]. S. Cho, K. A. Quilico Cousins et al. [103] compared digital speech and language features of tAD or LvPPA-affected patients in a biologically confirmed cohort and correlated these to neuropsychiatric test scores and CSF analysis results. Carrying out this study, they hypothesized that lvPPA cases would produce a speech characterized by more accentuated disfluency along with a more restricted vocabulary than the tAD cases. Furthermore, it was seen that tAD- and lvPPA-affected individuals share some linguistic abnormalities, for example, decreased speech production. E. Da Cunha, A. Plonka and colleagues [104] even went so far as to state that acoustic analysis would represent a clinically efficient alternative to refused lumbar punctures. They also claimed that “it offers the possibility to facilitate early, specific, and accessible neurodegenerative diagnosis and may ease early care with speech therapy, preventing the progression of symptoms”. For their study, eight AD and eight lvPPA people (for whom cerebrospinal fluid biomarker profiles were determined) were recruited. The participants attempted to perform a sentence repetition task that allowed an evaluation of potential lvPPA phonological loop impairments. They reported that temporal and prosodic markers significantly differentiate lvPPA and AD clusters at an early stage of the pathologic progression. LvPPA with an AD biomarker subset shows an acoustic pattern corresponding to an atypical AD form with a specific alteration of the phonological loop. In contrast, lvPPA without an AD biomarker subset presents with an acoustic profile similar to that of FTLD. Starting from 28 PCA- and 27 LvPPA-affected patients’ identification, J. L. Whitwell, P. R. Martin et al. [105] proved that PCAs have a slower progression than LvPPA and identified no relationships between the principal components analysis and age, sex, disease duration, amyloid PET findings, or apolipoprotein genotype.

### 2.7. Behavioral Variant/Dysexecutive Variant Alzheimer’s Disease

#### 2.7.1. The Definition History Helps to Clarify Clinical and Neuropathological Features

The term “Frontal AD” was coined in 1999 by Johnson et al. [106] to describe three intriguing cases which manifested predominant and early executive deficits and presented with Aβ and NFT pathology. Subsequently, several studies shed light on an AD dysexecutive phenotype (dAD) [107,108,109,110,111,112], but many of these did not include AD patients who had been previously confirmed through autopsy or biomarkers. Importantly, other postmortem [113,114,115,116], clinical [117,118], and case report [119,120,121] studies demonstrated that frontal AD could also present with early personality and behavioral changes (i.e., disinhibition, apathy, or compulsiveness). R. Ossenkoppele, Y. A. L. Pijnenburg et al. [122] proposed to adopt the definition “the behavioral/dysexecutive variant of Alzheimer’s disease” in place of “Frontal AD” in light of a relative sparing of frontal grey matter compared to the temporo-parietal regions in voxel-based morphometry analyses resulting from their own experience and other case reports in the literature. The frontal lobe is traditionally considered to constitute the regulatory core of behavior and executive functioning [123,124]. Thus, the authors presented various hypotheses about what pathogenic mechanism could underlie a predominant frontal grey matter destruction. For example, similar neuropsychiatric symptoms may be explained by vascular damage in frontal white matter, which leads to fronto-parietal disconnection [125], or by basal ganglia lesions which affect fronto-subcortical circuitries [126,127]. Both the former (Kim et al., 2013 for bvAD [128], Sjobeck et al., 2010 for dAD [129]) and the latter [125,127] pathological scenarios have been associated with both AD neuropsychiatric symptoms and executive dysfunction. Still, in their latest publication [130] focused on the same topic, the authors continue to question whether behavioral and dysexecutive represent two distinct entities or two steps within a continuum, two sides of the same coin. 

#### 2.7.2. Details about Population Distribution, Clinical Signs and Symptomatology

With these two variants, we draw closer to the frontotemporal dementia spectrum; they likely represent the most overlapping zones. The behavioral and dysexecutive variants account for only 2% of AD cases [84]. However, according to several articles [131,132,133,134], between 7 and 20% of clinically diagnosed FTD patients are found to have an underlying AD pathology. Meanwhile, according to [115,135,136,137] or post mortem evaluations [115,131,135,138], 10% to 40% of clinically diagnosed bvFTD patients test positive for AD biomarkers and/or display neuropathologically confirmed AD. However, the most widely used tau PET tracers ([18F] flortaucipir, [18F] MK6240, and [18F] RO948) bind selectively and with high affinity to AD tau aggregates (for example, combinations of 3R/4R tau in paired helical filaments). In the meantime, neocortical tau PET ligand retention in sporadic bvFTD is negligible. Thus, Tau PET serves as an excellent discriminator (with good accuracy) between AD and bvFTD. A total of 60% of bvAD affected individuals and 40% of dAD ones carry at least one APOE ε4 allele according to a retrospective study [122]. BvAD phenotype typically begins at a young age (mean [SD] age: 62.0 [7.3] years at diagnosis). This syndrome affects men more frequently than women (61.7% vs. 38.2%), in line with bvFTD but in contrast with tAD [139], and involves a lower frequency of APOEε4 carriership compared to tAD (47.5% vs. 66.1% [140]). Behavioral variants share many characteristics with the FTD behavioral variant (bvFTD) [111]. A recent retrospective study [141] showed that the first symptom of bvAD might be a typical obsessive-compulsive disorder (OCD) due to anterior cingulate network dysfunctions. It is well known that this syndrome begins with FTD typical personality changes, i.e., disinhibition, lack of empathy, disregard for societal norms, and sometimes hyperorality. In addition, affected individuals can exhibit delusions and hallucinations which are seldom the case with bvFTD patients. A very relevant work was recently published by V. Laganà, F. Bruno et al. on Neuropsychiatric or Behavioral and Psychological Symptoms of Dementia (BPSD) in bvFTD and AD (674 clinical records of bvFTD patients and 1925 AD patients from 2006 to 2018) [142]. In addition to recommending to read this work, we would like to highlight some fundamental aspects that emerged from it. Firstly, in the entire study population, BPSD are manifested in about 90% of affected individuals over the course of their disease. Secondly, in AD and bvFTD, mood disorders (such as depression and anxiety) occur before the other BPSD, with the same prevalence. Thus, mood disorders can serve as a “red flag” for detecting dementia. Moreover, these findings confirm the previously reported retrospective study about OCD. A systematic review and meta-analysis [130] reported that bvAD presented more severe neuropsychiatric symptoms in addition to those behavioral deficits compared to tAD at the time of diagnosis. Furthermore, two bvAD neuroimaging phenotypes could be distinguished (observed across reported bvAD cases): (1) a more AD-like pattern characterized by relative frontal sparing, and (2) a more bvFTD-like pattern presenting with both posterior and anterior involvement [111,118,122,136,143,144,145,146,147,148,149]. These patterns specifically affect certain brain networks (such as the salience network) which are activated when processing socioemotional information [150]. The former pattern is the most prevalent. Finally, R. Ossenkoppele, E. H. Singleton et al. [130] proposed that these phenotypes manifest themselves on a continuum. BvAD patients exhibited more severe behavioral symptoms than tAD and less severe compared to bvFTD. Regarding executive performance, it is worse in bvAD compared to tAD but better when compared to bvFTD, while no significant differences in memory performance were identified (even with tAD), even though bvAD showed a trend towards worse memory performance compared to bvFTD. Hence, although bvAD shares most pathophysiological characteristics with tAD, it shows a major clinical similarity with bvFTD. Compared to bvFTD, bvAD cases present less frequently with compulsive behaviors, but no differences were observed in disinhibition. Using the Neuropsychiatric Inventory (NPI), bvAD-affected individuals more frequently display agitation, delusions, and hallucinations compared to bvFTD patients. Both syndromes are associated with a relative frontal sparing and more posterior than anterior atrophy. As mentioned above, tAD patients seem to manifest neuropsychiatric symptoms more frequently (in terms of prevalence) than bvAD ones, even if bvAD-affected patients show more severe symptoms. It is likely that the bvAD concept has to be redefined because an overlap exists between bvAD and FTD on the basis of clinical presentation, cognitive deficits and neuroimaging results [143]. On the other hand, bvAD and tAD are likely to represent different clinical presentations of the same disease and not two different pathological entities. In fact, the available evidence suggests that they share common pathophysiological mechanisms and they are caused by the same pathogenic molecules [130]. Thus, it is conceivable that bvAD and tAD are part of the same disease spectrum, with the former being at one end and the latter at the other end of the spectrum. In conclusion, the neuropathological overlap between bvAD and tAD, together with their clinical similarities, supports the hypothesis that bvAD and tAD should be considered the two extreme phenotypes of the same disease spectrum. The cognitive and behavioral features of bvAD overlap with those of FTD, making it a challenging clinical diagnosis. The establishment of clear clinical criteria for bvAD, together with the implementation of new diagnostic tools and techniques, is essential for the accurate diagnosis and management of patients with this complex clinical syndrome. The recent development of biomarkers and other diagnostic tools for AD, including tau PET tracers and blood-based biomarkers, will certainly contribute to improve the diagnosis and treatment of bvAD. Further studies are necessary to validate these preliminary findings and to establish clear diagnostic criteria for bvAD. These advances in our understanding of bvAD will hopefully lead to improved patient care and potentially to the development of new therapeutic strategies for this condition. 

#### 2.7.3. Neuroimaging Findings Permit a More Specific Clusterizations of Patients

N. Corriveau-Lecavalier, M. M. Machulda et al. [151] described a case series of six dAD patients in which mean age at diagnosis was 57.3 years, and patients annually underwent a clinical, multimodal imaging and cognitive evaluations for a mean period of 3.7 years. Authors divided these cases into three subtypes on the basis of their FDG-PET hypometabolism profile (with almost completely overlapping tau distribution pattern): (1) predominantly left parieto-frontal (ldAD); (2) predominantly right parieto-frontal (rdAD); (3) predominantly biparietal (bpdAD) (two patients for each subdivision). Notwithstanding that prominent executive deficit was highlighted in all patients, several differences were observed between these three subsets. ldAD cases showed the greatest deficit in measures of verbal working memory and verbal fluency. On the other hand, rdAD patients presented with more severe alterations in visual skills than language-related domains and committed more perseverative errors on a cognitive flexibility measure. Instead, bpdAD-affected individuals showed predominant cognitive flexibility, inhibition deficit and a slower clinical course with relative sparing of working memory. None of the ldAD cases manifest BPSD, unlike the other two subgroups. Also, in the behavioral/dysexecutive variant, as could be expected, amyloid shows a widespread pattern. Structural imaging may present with remarkable frontal atrophy, which can be asymmetric [152]; medial temporal regions instead may be relatively spared [151,152,153,154,155,156,157]. In about 50% of cases, FDG-PET reveals frontal and parietal hypometabolism [158]. Some researcher groups have reported that hypometabolism localizes more to the medial frontal or dorsolateral frontal regions than the orbitofrontal regions, as it happens in bvFTD [159]. Behavioral variant AD shows a frontoinsular hypometabolism of greater extent but similar temporoparietal hypometabolism in respect to tAD [158]. Various studies in the literature with the aim to assess glucose metabolism with [18F] FDG-PET or perfusion with SPECT (seven studies for a total of 88 participants) in bvAD also showed heterogeneous results, which range from a predominantly temporoparietal hypometabolic pattern [111,146,147,149] to a predominantly frontal pattern [160] or mixed frontal and temporoparietal patterns [161,162,163,164]. Surely, the most hypometabolic areas in dysexecutive AD are the middle temporal, inferior temporal and angular gyri [91]. In frontal variant AD, using PET to trace tau pattern distribution, a great tracer retention in the frontal lobes in addition to the classical medial, lateral, inferior temporal and lateral parietal ones [165] has been obtained. In bvAD and tAD, Am-yloid-PET studies (two studies [146,160] for a total of 28 involved individuals) reveals almost the same Aβ burden and distribution. Regarding tau-PET, we can consider two studies for a total of 22 participants. One [146] showed a temporoparietal profile characterized by an anterior regions higher uptake in bvAD than tAD, whereas the other [166] reported heterogeneous profiles across bvAD-affected individuals. Several Structural MRI studies (16 studies; 92 participants) have reported temporoparietal-predominant [122] frontotemporal-predominant and insular-predominant [143,144,145,149] or frontoparietal-predominant [146] atrophy patterns for patients with bvAD. In three studies [122,146,149], no difference was observed between bvAD and tAD, and a moderately greater involvement of frontal regions in bvAD in respect to tAD was observed in three other studies [143,144,145]. Other neuroimaging analysis reported a major cortical thinning in bvAD than in bvFTD in left temporal-occipital areas, while in bvFTD cortical thinning is more accentuated in left inferior frontal cortex in respect to bvAD, whereas the latter showed thinner prefrontal and anterior temporal areas compared to tAD. BvAD patients had higher average volume than tAD-affected individuals in posterior hippocampus and bvFTD-affected individuals in anterior hippocampus (if the values were previously adjusted for age and intracranial volume) [167]. We can also report some studies [143,144,160] which yield recent findings about functional connectivity, and another study which was dedicated to white matter hyperintensities [149] in bvAD. In the recent past, several researchers suggested a possible significant role played by oxytocin in dementia social-emotional symptoms. However, E. G Johnson, W. Kuiper et al. [168] demonstrated no association between oxytocin plasma levels, social behavior or emotion processing in patients affected by bvFTD, AD or Semantic dementia (SD), conducting a study across 30 bvFTD, 39 AD, 28 SD and 24 healthy controls. After blood samples were collected in order to assess the hormone plasma range level, careers had to complete the Cambridge Behavioral Inventory and the Neuropsychiatric Inventory (for symptomatologic evaluation). Regarding possible bvAD/dAD co-pathologies, in the above-mentioned retrospective study [122], the following proportions were reported: 42% of Lewy body disease, 25% of cerebrovascular disease and 44% of argyrophilic grain disease; values that fall within the previous reports on SAD (sporadic AD) range [169,170,171,172]. On the other hand, cerebral amyloid angiopathy (CAA) is present in 64% of bvAD/dAD cases, i.e., less frequent than the percentage found in earlier neuropathological studies in AD, in which it was close to 90% [173,174]. Finally, argyrophilic thorny astrocyte clusters in three patients were found [175]. 

### 2.8. AD with Corticobasal Syndrome (CBS)

#### 2.8.1. Definition and Milestone Studies

Corticobasal syndrome usually occurs due to corticobasal degeneration (CBD), a four-repeat tauopathy. However, various neuropathological studies have noted that 15% to 54% of cases are associated with AD pathogenesis [131,176,177,178]. Similar to other atypical AD phenotypes, CBS is tied to a hippocampal sparing pattern [29]. We can trace a brief history of the CBS spectrum beginning with a study by Boeve et al. They investigated the postmortem neuropathology of 13 clinically suspected CBD cases and discovered that only seven patients (54%) showed neuropathological findings of CBD [179], while two presented with ADNC and others with progressive supranuclear palsy (PSP), Pick’s disease, Creutzfeldt–Jakob disease (CJD), and non-specific degenerative variations. They maintained that characteristic clinical presentations were explained by the topographical distribution pattern of the lesions, regardless of the underlying disease. Afterwards, Cordato et al. carried out a study on a cohort of six affected individuals, four with CBD and two with PSP, and coined the term “corticobasal syndrome” (CBS) to encompass their shared clinical features [180]. Finally, Boeve et al. proposed that CBD should be limited to the neuropathological disorder, while CBS could describe the clinical syndrome of progressive asymmetric rigidity and apraxia [181]. 

#### 2.8.2. Clinical Features

AD-CBS is characterized by more pronounced motor cortex atrophy, more accentuated substantia nigra neurodegeneration, and greater motor cortex Tau accumulation compared to tAD [182]. According to some studies, given that biomarker analysis can be invaluable in the context of CBS, a careful neurological examination may be critical to avoid misdiagnosis [18]. Other authors, however, suggest that clinical features have a low predictive value for underlying pathologies [183], and molecular biomarkers that can reveal specific dysfunctional proteins show promise for improving antemortem diagnoses [182]. The current diagnostic criteria rely on three major features: akinetic-rigid syndrome, limb apraxia, and cognitive impairment, along with focal or segmental myoclonus, limb dystonia, alien limb phenomenon, cortical sensory loss, and dyscalculia as minor criteria [184]. In 2011, Zhao Y. et al. [185] studied 43 patients and reported that longer disease duration, younger age at onset, memory impairment, dressing apraxia, hemi-sensory neglect, visuospatial problems, and myoclonus, but not Gerstmann syndrome, are more commonly associated with CBS-AD than CBS-CBD. No differences were found in the frequency of aphasia, limb apraxia, pyramidal motor signs, tremor, parkinsonism, dysphagia, alien limb phenomenon, dysarthria, frontal release signs, gait disorders, and postural instability. However, extraocular disturbances and rigidity were more prevalent in the CBS-CBD cluster. The age of onset for CBS-AD is typically younger than for CBS-CBD [186]. In 2016, Lee CYD et al. [187] conducted an analysis of 45 CBS-affected individuals, considering CSF values of Aβ and tau proteins. They reported that myoclonus and Gerstmann syndrome are more frequent in CBS-AD. Specifically, myoclonus was more frequent in AD-CBS than CBD-CBS in one study [186]; however, other research groups obtained different findings [183,188,189]. There are also conflicting results regarding tremor. According to one study, it was more frequent in CBD-CBS than in AD-CBS, but another study found no significant difference. Only in one work [188] reported the frequency of visual neglect compared, where it was more common in AD-CBS. Diagnostic concerns differ for affected cognitive domains in relation to neuropsychological examination due to variable phenotypic presentation. Deficits in tests of speech and language (especially speech apraxia evaluated by speech-language pathology), anomia (e.g., as highlighted by naming impairment on the Boston Naming Test), and visuospatial anomalies (particularly in construction, as measured by the WAIS-IV Block Design and Rey-Osterrieth Complex Figure Test) are more common. Other less represented symptoms that may manifest include impaired executive function (specifically set shifting, problem-solving), perseverations, and difficulties with sequences. 

#### 2.8.3. Neuroimaging Findings

The widespread pattern of brain amyloidosis and cortical tau deposition (usually more accentuated in the hemisphere contralateral to the affected limb), often without sensorimotor cortex sparing [88] or low CSF amyloid-β combined with high total tau and phosphorylated tau, tend to indicate CBS-AD. Toledo et al., in analyzing antemortem AD-related CSF biomarkers in 142 autopsy cases (including PSP and CBD), noticed that the AD pathology cluster could be discriminated from the group without AD pathology with a 96% sensitivity and an 87% specificity [190]. However, even if the CSF biomarkers show an AD profile, this does not exclude other pathologies [182]. Structural neuroimaging reveals more pronounced contralateral frontoparietal neurodegeneration in relation to the affected limb [18]. The lateral temporal lobe could also be involved [177,184,185,188]. A meta-analysis of amyloid PET studies [191] reported that 23 out of 61 (38%) individuals affected by CBS showed amyloid positivity on PET, and this frequency decreased with age. CBS is strongly linked to hippocampal-sparing AD, which typically affects younger people compared to other AD subtypes [29]. While a positive Aβ PET is not sufficient to confirm AD pathology as the primary cause and it may be necessary to combine this finding with other biomarkers, primarily tau PET [192,193], when negative, Aβ PET allows ruling out the presence of an underlying AD pathology [182]. Ali F. et al. [194] assessed regional profiles of [18F]flortaucipir uptake in 14 CBS patients. Among these, six were Aβ-PET-positive, which included three patients that showed pronounced [18F]flortaucipir uptake across many cortical regions, consistent with AD-CBS. Notably, negative Aβ PET patients who initially presented with apraxia of speech demonstrated heightened [18F]flortaucipir retention in the supplementary motor area and precentral cortex, while CBS patients without apraxia of speech did not show significant retention. By means of in vitro autoradiography using non-AD tauopathies brains, it was observed that there is less binding affinity of [F18F]AV-1451 to 4R tauopathies than to AD145 [195]. Thus, [18F]flortaucipir PET may not be reliable in detecting non-AD tauopathies [196,197]. [18F]PI-2620, a “second-generation” tau PET tracer and an analogue of [18F]flortaucipir, shows a high affinity for NFT with low off-target binding towards amyloid, monoamine oxidase (MAO)-A, and MAO-B [198]. In Palleis C. et al. [192], positive [18F]PI-2620 retention was observed in 91% of Aβ-positive CBS and 65% of Aβ-negative CBS, while only 7% of controls showed positive [18F]PI-2620 retention (93% specificity). The putamen and external segment of the pallidum were also reported as the most common regions in the CBS group for retention. Retention in cortices was highest, and more likely in Aβ-positive CBS compared to Aβ-negative CBS. Specifically, the dorsolateral prefrontal cortex was the most common area with retention. A diffuse atrophy profile has been suggested to indicate an underlying AD pathology. Moreover, individuals with CBS-AD often exhibit asymmetric posterior hypometabolism or hypoperfusion of the lateral temporal and lateral and medial parietal lobes and the posterior cingulate area [199]. In a very small cluster of cases, CBS is familial due to early-onset AD-associated mutations, such as PSEN1 and APP [200,201,202,203].

## 3. A Sea of Atypical Form Classifications: Let Us Learn to Swim in It!

Light does not reach the depths of this sea; thus, anyone who wants to understand something new about atypical forms find themselves playing a blind man’s buff. Which classification should we use? What is the best criterion? The literature suggests that Alzheimer–Perusini’s disease is a continuum [18], manifesting itself in a wide spectrum of different phenotypes, likely based on a unique original molecular mechanism that becomes altered along with an induced or inducing cellular anomaly. Hence, how can we interprete the numerous and detailed descriptions provided so far? Until today, it represents the closest possible scenario to the truth while we wait for our Pygmalion-DMT. All these classifications are useful in the clinical setting, where distinguishing between various syndromes is important in aiding patients to live with the disease and in trying to slow its progression. According to Polsinelli et al. [18], differentiation can primarily be assisted by identifying the most prominent symptoms and the timeline of their onset. Nevertheless, novel neuroimaging techniques and the ability to combine and analyze a large amount of data are significant aids in achieving this goal. In addition, classifications have an inherent educational value. 

## 4. Transforming the Sea into a Bathtub: A Genetic–Molecular Attempt

We pursue this goal by first presenting genetic classifications, followed by a discussion on the involvement of genomics and other omics sciences in this field. The Religious Orders Study and Memory and Aging Project (ROSMAP) recruited 222 AD-affected individuals who underwent molecular profiling and gene expression analysis in order to cluster them based on a functional molecular criterion. In this way, two pathologic subsets were identified: synaptic and inflammatory. The former is more associated with APOE ε3/ε4 genotype carriers and males, characterized by disrupted synaptic vesicle priming and plasticity. The latter is typically associated with homozygous APOE ε3 carriers and is more prevalent in women due to dysfunctional inflammatory pathways such as IL-2, IFN-α, and γ [62]. In 2021, Sorrentino et al. [204] demonstrated that a molecular-based AD patient stratification is possible starting from 25 brain levels of phlogistic factors. They identified three subsets. AD-cluster 1 (AD-CL1) was characterized by a predominance of Innate Immunity Factors (IIFs), with high levels of CD14, a co-receptor involved in microglia-mediated Aβ clearance. This cluster displayed strong microglial activation, the lowest number, and the largest size of Aβ deposits, suggesting a functional Aβ clearance. However, AD-CL1 had both the earliest age at onset and age at death. In AD-CL2, all inflammatory factors were elevated, with no specific neuroinflammatory molecular family identified, and the highest levels of cytokines and chemokines present. The other features of AD-CL2 included the highest levels of Aβ42 and Aβ40 species in both soluble and insoluble fractions of brain homogenates, the highest age at onset, the fastest disease progression rate, and the lowest Braak stage. These characteristics indicate a pronounced Aβ deposition trend, leading to derangements in the neuropil, neuroinflammation, and tauopathy, with an aggressive clinical phenotypic manifestation. AD-CL3 appeared to have a low neuroinflammatory profile, probably due to minimal microglial activation. This cluster had the lowest amount of almost all the molecules tested, except for MMP-8, MMP-9 (the MMP family was the most represented in this cluster), CX3CL1, and LCN2. Finally, this subgroup had low levels of Aβ in the brain and the longest disease span. R. A. Neff, M. Wang et al. [205], using a network-based clustering algorithm called WSCNA (weighted sample gene network analysis), identified five subsets among the molecular subtypes of AD in the Mount Sinai/JJ Peters VA Medical Center Brain Bank (MSBB-AD) clusters across 151 chosen individuals with parahippocampal gyrus (PHG) transcriptomic data (clusters A, B1, B2, C1, and C2). These five subsets can be further grouped into three main classes: A, B (which contains B1 and B2), and C (which contains subtypes C1 and C2). They observed strong upregulation for Aβ binding, clearance, and fiber formation pathways in C1, and scavenger receptor activity in C1 and C2, unlike A and B1, where these pathways are downregulated. Additionally, “GNF2_MAPT” pathway-related genes showed significant upregulation in A, B1, and B2 but a downregulation in C1 and C2, while tau protein binding and tau-related P35 pathway genes were upregulated in A. Neurotransmitter levels showed a distinct trend. Broadly, glutaminergic, γ-aminobutyric acid (GABA), glycinergic, and dendritic synaptic-related pathways were down-expressed in C without any variations in cholinergic and dopaminergic synaptic pathways (probably selectively resilient to AD subcluster molecular changes). The same synapse pathways presented with strongly increased expression levels in A and B, except for the glycinergic synapse that was found to be upregulated only in A. This expression profile corresponds to synaptic excitation pathway differences among various subgroups, i.e., excitatory synapses are upregulated in classes A and B but downregulated in class C. Findings about the immune response are also very intriguing. Immune-related pathways (for instance, the innate and adaptive immune response, inflammatory state, immune system activation, endothelial cell migration, and circulatory system development) are upregulated in comparison to healthy controls in B2, C2, and especially in C1, but they are downregulated in A and B1. This upregulated expression pattern coincides with increased expression of blood–brain barrier, basement membrane, and cell matrix adhesion genes. Several molecular pathways are found to be involved exclusively (or almost exclusively) in a specific cluster. Elevated levels of ubiquitination and polyubiquitination, protein catabolism, proteasome, proteins targeting for destruction, and other protein degradation-related genes were found in A. On the other hand, acid secretion, acidic amino acid transport, and other organic acid-related genes were upregulated in B. It was previously demonstrated that dysfunctional lysosome acidification in neurons correlates with synaptic AD destruction and neurodegeneration as well as diminished long-term potentiation [205]. Tijms et al. [206] used CSF proteomics as a criterion to classify three pathophysiological subsets (1) characterized by neuronal hyperplasticity; (2) with innate immune activation; and (3) relying on a blood–brain barrier alteration. These different phenotypes appeared before the Aβ pathology manifested (reached an abnormal amount). Hence, in the future, new early biomarkers might be discovered through proteomic subsets, or more likely, new metabolomic profiles will be recognized from which to deduce new early biomarkers. The last omics in a hypothetical timeline is probably mitoepigenetics (involving D-loop alterations and a still partially hidden world of ncRNAs), which could shed light on the AD spectrum [207,208]. Investigating beyond the recent acquisitions in the field of omics sciences falls outside the scope of this review. Familial AD cases (FAD) are EOAD forms, which can be distinguished into Mendelian and atypical ones. Thus, regardless of preferredclassification, the AD spectrum ranges from Sporadic AD (SAD), where probably thousands of possible associated polymorphisms exist, besides epigenetic alterations that transform normal aging into a pathology, to mAD, in which a single mutated gene (APP, PSEN1, or PSEN2) is responsible for the etiopathogenesis. The core of this spectrum is represented by atypical forms, which are probably characterized by a small series of associated genes along with environmental influence. Some mutations for atypical forms are known. Here, we report, as an example, SEMA3C, CTNAP5, and FAM546A [17] that were found to be related to PCA-AD. Therefore, understanding the etiopathogenesis of atypical forms by the omics approach allows recognizing the highly complex web of interconnections that regulates AD’s pathogenesis and it provides the possibility of developing new AD early diagnosis methods and novel therapeutic strategies, as it is outlined in the following paragraphs.

## 5. Transforming the Sea into a Bathtub: An Immune Attempt

**Figure 6:**
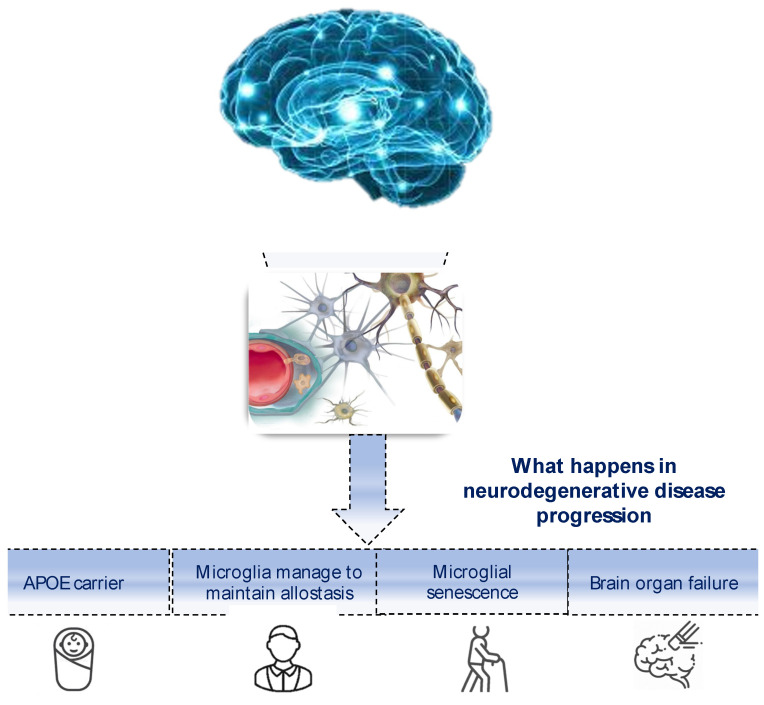
Immunopreoteostasis theory [211].

“CNS is an immunological sanctuary” has been taught for decades to thousands of diverse students, primarily due to the apparent absence of adaptive immunity. However, neurodegenerative progressions often present with alterations of the BBB, as extensively acknowledged [209], are considered one of the main features of these diseases. Hence, under these particular conditions of permeability, the concept of what can or cannot enter the brain is distorted. Today, we know we must discuss the Psychoneuroimmunoendocrine (PNEI) system, in which the immune [210] and endocrine system appear to play a key role connecting the brain and the rest of the body. As for the brain, microglia and astroglia constitute a link between the brain and systemic immunity. Delving into the specifics of neurodegenerative disorders, McFarland and Chakrabarty [27] recently dedicated a review to glial cells in Alzheimer–Perusini’s disease, starting from the assumption that it is not a neuron-predominant illness. In Karran E. et al. [211], a new and innovative (due to its shift of perspective) etiopathological theory was proposed (Figure 6). Microglia act as a guardian of protein and ion homeostasis, thus normal cognitive aging changes could stem from its senescence and further neurodegeneration from exacerbation of senescence effects. As for early onset forms, a genetic trigger appears to be the primary suspect for glial dysfunction, especially the APOE genotype (thus integrating genetic and immune responses into a continuum). This hypothesis relies on the newly acquired concept of immunoproteostasis [19,34], which results in a dynamic stationary equilibrium of what is secreted and what is eliminated, with alterations leading to proteinopathies [212]. From these assertions, it emerges that likely microglia follow various activation phases which correspond to AD stages. In Prokop S et al. [213,214], starting from histopathological evidence, it is shown that microglia increase cell numbers during early AD stages and then decline in advanced disease phases. Considering several radiation biology studies showing various linear dose–response theory limitations [215], microglia seem to act according to a hormetic response based on AD proteinopathies and neurodegeneration allostatic variations. Beyond APOE, we can mention other genes related to microglial dysfunction in AD: ABCA7, ABI3/NESH, ADAM10, ALPK2, BIN1, CASS4, CD33, CLU, CR1, HLA-DRB1, INPP5D/SHIP1, MS4A6A, PICALM, PLCG2, SORL1, SPI1/PU.1, TREM2 [27] and LILRB2 [216] (we suggest consulting www.brainrnaseq.org, last accessed on 15 June 2023, and Zhang Y. et al. [217] for better insight into human cell type expression, AlzPedia (Alzforum.org) and Hansen DV et al. [218] for direction and putative function of these genes). Once just two (M1 and M2, like macrophages) microglia subsets were considered; however, now is the single-cell information era, hence we were able to appreciate a series of microglial activation states; or, possibly, it is better to talk about a range of intermediate phenotypes, which are brain-area- or context-specific. As stated above, these different guises in which microglia appears correlate with different physiological and non-physiological conditions [219,220,221,222]. In Masuda T. et al. [222], it is hypothesized that glial dysfunction may lead to tauopathy and neuritic plaques which in turn induce neurodegeneration, although the exact underlying mechanisms remain unknown, as an elusive deus ex machina. This finding could be corroborated by various studies demonstrating the possibility that an upstream inflammatory condition may precede Aβ plaque formation [132,223,224]. In [27] by McFarland, it was claimed that lowering NLRP3 inflammasome activity also reduces the Aβ burden in APP/PS1 mice. Nevertheless, other studies reported evidence contradictory to this theory because of the proven microglia capability of Aβ phagocytosis [19,154,225,226,227]. Indeed, microglia represent the “needle of the balance” in these situations. Astro- and microglia produce most of the (strongly immunogenic) molecules that make up pathological accumulations [27]. What emerges from this is the speculation that these immunogenic molecules are secreted with a precise role in allostasis maintenance; thus, when microglia become dysfunctional, this fine adjustment gradually goes wrong (recalling the previously mentioned concept that depicts microglia as brain allostasis guardians). Different brain-area-specific microglia phenotypes, when altered, may induce a determinate symptomatologic spectrum, explaining the high number of different atypical forms. In Deleidi M. et al. [228], senescent microglia were described with a series of peculiar features ranging from telomere shortening to lipofuscin accumulation, from decreased arborization, motility, and phagocytic activity to augmented inflammatory cytokine secretion and abnormal upregulation of MHC-II molecules. Article [229] correlates this type of microglia with iron dysallostasis. Microglial senescent-associated variations have been observed anticipating NFT formation. TREM2 mutations carriers, among AD-affected individuals, present with a great amount of dystrophic microglia [213] compared to similar-AD-neuropathology-affected non-carrier people, suggesting a major vulnerability for senescent AD risk gene carrier microglia. Moreover, other dysfunctional microglial subsets were characterized by means of ultrastructure or transcriptomics differences [222,230,231]. For example, “dark microglia” [232,233,234] were described. It owes its name to a characteristic condensed and electron-dense nucleoplasm [233], without evident apoptotic or necrotic lesions. These peculiar cells make tight contact with vessels and synaptic clefts; hence, it is simple to imagine their involvement in the hematoencephalic barrier (HEB) and the synaptic function [230]. In a tauopathy model, senescent micro- and astroglia were identified and a senescence regulatory pathway was determined, i.e., GAS-STING, which is related to immune activation, too [235]. It represents an immune activation and glial senescence linking bridge during progressive AD neuropathology. Other microglial phenotypes were identified by means of transcriptomic analyses, in particular single-cell (sc) [236,237], single nucleus (sn) [238], and spatial [239]. In AD cases, many diverse phenotypes were recognized, all closely bound to Aβ plaques or neurodegeneration regions [222]. Microglial response to molecular pathology is not uniform; every region-specific phenotype responds in a unique manner [240]. We can distinguish a homeostatic type of microglia (HM) that comprises two subsets (H1M and H2M that are usually almost equivalent in number [241,242,243]) and is engaged in hunting for insults or injuries based on TGF-β signaling [244,245]. Inflammatory state-associated or responsive-to-injury microglia usually present with a decreased homeostatic gene expression profile [27]. TMEM119, P2RY12, and CX3CR1 are common in both H1M and H2M—evidence of a partial overlap in gene expression. Unlike H1M, H2M may be more sensitive to aging since their numbers are slightly lowered in older mice and could be primed in order to allow a more rapid response to an activated state [242]. From single-cell sequencing analyses performed on healthy and AD brains, a protective microglial subset was identified, which was called disease-associated microglia (DAM) [246]. It appears to be triggered by a two-step (TREM2-independent and TREM2-dependent) sequential transduction mechanism. The former starts with a downregulation of allostatic genes, such as the above-mentioned TMEM119, P2RY12, and CX3CR1, along with an augmented expression rate of AD-related ones, for instance, APOE, CTSB, and CTSD. The latter assumes an increased expression rate for phagocytosis and lipid-metabolism-related genes, for example, CST7 and LPL. In a mouse AD model, a close relationship between these microglial subpopulations and Aβ plaques was observed; nevertheless, a role in deposits remodeling was also supposed for them. It is likely that these DAM-AD connections are not suggestive of specificity, since the same subset was observed in ALS, FTD, and CK-p25 mice [246,247]. However, it is more probable that there are no disease-unique phenotypes (the following reported phenotypes information confirm this assertion), but just the above-mentioned brain-area-specific subtypes. Moreover, in ALS, AD, and multiple sclerosis mouse models, another relevant microglia cluster was found: MGnD [248]. It proliferates as a response to neuronal degeneration/apoptosis (or, more likely, necroptosis [249]) by means of the TREM2-APOE pathway in combination with TGF-β suppression, which is involved in homeostatic microglial regulation. MGnD gene expression feature is a 28-pro-inflammatory gene upregulation in the general context of a decreased rate of homeostatic gene expression. In a snRNAseq analysis [242], four microglial subsets were identified: activated response microglia (ARM), interferon response microglia (IRM), transiting response microglia (TRM), and cycling and proliferating microglia (CPM). Both ARM and IRM were primed in normal aging, but only ARM responded to Aβ accumulation. Genes related to the innate immune response and interferon response pathways constitute the driving transcriptional characteristics of IRM. ARM mainly expresses inflammatory genes, MHC-II molecules, and tissue regenerative ones, among which is a gene cluster that includes, for instance, APOE and TREM2, overlapping with GWAS, previously identified AD genes. TRM shows the same transcriptional pattern as ARM, just at lower levels, while CPM represents a very small subgroup in respect to the whole microglial population. In physiological aging, 10% of microglia is constituted by ARM; however, a great proliferation of this subcluster was observed when Aβ pathology was developing. Depleting APOE impedes ARM response to Aβ, but not the IRM reaction. McFarland KN et al. [250] investigated transcriptional variations in response to different Aβ conformers in primary cultured microglia and then compared the resulting values to a total RNA-seq dataset performed on APP transgenic TgCRND8 mice of various ages. Treating microglia in vitro with different Aβ conformers (fibrillar, monomeric, or oligomeric) triggered diverse and specific microglial phenotype transformations. MGnD (proved by the transcriptional pattern [246]) increased after all treatments, whereas DAM [248] increased with fibrillar but not with oligomeric Aβ42. As we await new insights into astrocytes phenotype variety and a better characterization of microglial ones, we can only hypothesize the mechanism underlying the atypical presentations of AD, step by step.

5. And If It Was Just a Glass, Would It Be Half Full or Half Empty? Closing the Circle

Having reached this point, we should wonder whether Alzheimer and Perusini’s other patients would have a better quality of life today. It is likely that they would. This possibility leads us to consider the glass half full, first because of the vast amount of molecular advances over the past ten decades or so. The molecular biology of the nervous system represents a marvelous world that is still little known, in which weak, stationary dynamic equilibriums constitute the basis of neural plasticity and therefore of our Pindaric flights of thought. By furthering our knowledge about these mechanisms, we will be able to unravel the natural history of dementia in general. We have already achieved the capability to perform ante mortem diagnoses, combining several innovative neuroimaging techniques (another field of science that has experienced rapid development in recent years), such as FDG-, tau-, Aβ-PET, various functional and structural MRI applications, SPECT for cerebral perfusion, etc. When we finally uncover the precise molecular mechanisms underlying neurodegenerative diseases, we will be able to design disease-modifying therapies. In the meantime, a wider range of information can still be useful for caregivers to improve their approach to patients. In addition, based on this information, innovative systems can be developed to exercise damaged brain areas and thus slow down the progression of the disease.

## Data Availability

Not applicable.
